# Psychometric Properties of the Functional Impairment Checklist (FIC) as a Disease-Specific Patient-Reported Outcome Measure (PROM) in Previously Hospitalized COVID-19 Survivors with Long-COVID

**DOI:** 10.3390/ijerph191811460

**Published:** 2022-09-12

**Authors:** César Fernández-de-las-Peñas, Maria Palacios-Ceña, Jorge Rodríguez-Jiménez, Ana I. de-la-Llave-Rincón, Stella Fuensalida-Novo, Margarita Cigarán-Méndez, Lidiane L. Florencio, Silvia Ambite-Quesada, Ricardo Ortega-Santiago, Alberto Pardo-Hernández, Valentín Hernández-Barrera, Domingo Palacios-Ceña, Ángel Gil-de-Miguel

**Affiliations:** 1 Department of Physical Therapy, Occupational Therapy, Rehabilitation and Physical Medicine, Universidad Rey Juan Carlos, 28922 Alcorcón, Spain; 2 Department of Psychology, Universidad Rey Juan Carlos, 28922 Alcorcón, Spain; 3 Consejería de Salud Pública, Comunidad de Madrid, 28009 Madrid, Spain; 4 Department of Public Health and Preventive Medicine, Universidad Rey Juan Carlos, 28922 Alcorcón, Spain

**Keywords:** function impairment checklist, function, COVID-19, long-COVID, validity

## Abstract

The severe acute respiratory syndrome coronavirus 2 (SARS-CoV-2) virus is associated with a plethora of long-lasting symptoms (long-COVID). The presence of long-COVID symptoms causes decreased functionality. This study described the psychometric properties of the Functional Impairment Checklist (FIC), a disease-specific patient-reported outcome measure (PROM) used for evaluating the functional consequences of SARS in previously hospitalized COVID-19 survivors with long-COVID symptoms. The LONG-COVID-EXP-CM is a multicenter cohort study including patients hospitalized with COVID-19 during the first wave of the pandemic in five hospitals in Madrid. A total of 1969 (age: 61 ± 16 years, 46.4% women) COVID-19 survivors with long-COVID completed the FIC at a long-term follow-up after hospitalization (mean: 8.4 ± 1.5 months). Internal consistency (Cronbach alpha value), reliability (item-internal consistency, item-discriminant validity), construct validity (exploratory factor analysis), floor effect and ceiling effect were calculated. The mean time for fulfilling the FIC was 62 ± 11 s. The Cronbach’s alpha values reflecting the internal consistency reliability were 0.864 for FIC-symptoms and 0.845 for FIC-disability. The correlation coefficient between the FIC-symptoms and FIC-disability scale was good (r: 0.676). The ceiling effect ranged from 2.29% to 9.02%, whereas the floor effect ranged from 38.56% to 80.19%. The exploratory factor analysis showed factor loadings from 0.514 to 0.866, supporting good construct validity. Women exhibited greater limitations in all physical symptoms and disability-related domains of the FIC compared with men (all, *p* < 0.001). Further, younger patients (those aged <45 years) self-reported lower physical symptoms and disability-related domains than older patients. In conclusion, this study indicates that the FIC has good psychometric properties to be used as a specific-disease PROM to measure function and disability in COVID-19 survivors with long-COVID.

## 1. Introduction

The coronavirus disease 2019 (COVID-19) was initially considered a respiratory pathology; however, a multisystemic affection is found in most patients [1]. This multisystemic affection explains the plethora of symptoms experienced at the acute phase of the infection and, particularly, after the acute phase, the so-called long-COVID [2]. The presence of long-COVID symptomatology can be present in up to 60% of individuals who had survived COVID-19 [3]. In fact, long-COVID is associated with worse health-related quality of life (HRQoL) [4], a decrease in daily living activities [5] and loss of work ability [6]. A recent definition of post-COVID-19 condition has included that function should be affected [7]: …these symptoms generally have an impact on everyday function… Accordingly, proper evaluation of function in individuals with long-COVID is essential.

Patient-reported outcome measures (PROM) consist of self-reported questionnaires evaluating aspects of a condition. PROMs can be generic—those evaluating symptoms present in several diseases—or disease-specific—those evaluating specific symptoms more associated with a particular condition. The Post-COVID-19 Functional Status Scale (PCFS) was created as a disease-specific PROM assessing the functionality of COVID-19 survivors with long-COVID symptoms [8]. The PCFS has shown good construct validity; however, it just consists of one question [9]. Due to the multisystemic affection and the heterogeneity presentation of long-COVID, the PCFS could not cover the entire spectrum of this condition; therefore, complementary specific-disease PROMs should be encouraged. 

The Hospital Authority Hong Kong developed a disease-specific PROM for evaluating the functional consequences of Severe Acute Respiratory Distress Syndrome (SARS): the Functional Impairment Checklist (FIC) [10]. The FIC has just been used in the original publication [10], and it has been never used again. The FIC has been found to be positively correlated with physical function and health-related quality of life in individuals who had survived SARS [10]. Due to the similarities between SARS and SARS-CoV-2, the FIC could be used as a disease-specific PROM in COVID-19 survivors suffering from long-COVID. In fact, the FIC mainly assesses physical symptoms such as breathlessness and fatigue, probably the most prevalent post-COVID symptoms [3]. This study aimed to describe the internal consistency, reliability and construct validity of the FIC in a sample of previously hospitalized COVID-19 survivors suffering from long-COVID. 

## 2. Methods

### 2.1. Participants

The LONG-COVID-EXP-CM is a multicenter cohort study including patients hospitalized due to SARS-CoV-2 infection from 10 March to 31 May 2020 in five urban hospitals in Madrid (Spain) [11,12,13,14,15,16]. All of the included participants were diagnosed with the real-time reverse transcription-polymerase chain reaction (PCR-RT) assay of nasopharyngeal and oral swab samples and positive radiological findings at hospitalization. All patients discharged from the hospitals were anonymously included in a database, and a selection of 400 patients from each one was conducted by a randomization software. The study was approved by the Ethics Committees of all institutions (Universidad Rey Juan Carlos 0907202015920, Hospital Universitario Infanta Leonor 092-20, Hospital Clínico San Carlos 20/495E, Hospital Universitario Fuenlabrada 1517, Hospital Severo Ochoa 5112020, Hospital Universitario Fundación Alcorcón 20/126). All participants provided their informed consent before entering into the study. Data from the LONG-COVID-EXP-CM study have been used in previous letters to the editor or publications [11,12,13,14,15,16], but the current data presented here are completely new and have not previously been published.

### 2.2. COVID-19 and Post-COVID Data Collection

Demographic data, clinical data and hospitalization data were collected from hospital medical records, as previously explained [11,12,13,14,15,16]_._ Additionally, a telephone interview was performed for each participant to systematically assess the presence of symptoms that have appeared after hospital discharge (post-COVID-19-related symptoms) from a predefined list (see Table 1). The participants were free to report any other symptom not included in the list, if present. We defined a post-COVID-19 symptom as any symptom that appeared after the SARs-CoV-2 acute infection (no later than the following month after hospital discharge) and that persisted at the time of the study [2,7]. 

### 2.3. Functional Impairment Checklist (FIC)

All participants fulfilled the Functional Impairment Checklist (FIC), an eight-item disease-specific questionnaire used for evaluating the functional consequences of SARS. Each item is evaluated in terms of four degrees of severity (0: no, 1: mild, 2: moderate, 3: severe).

The first four items assess symptoms including breathlessness at rest, breathlessness on exertion, general fatigue and muscle weakness, and their sum forms the symptom-based impairment score (FIC symptoms). The remaining four items assess physical limitations in occupational daily living activities, leisure/social activities, basic daily living activities and instrumental activities of daily living as a result of the effects of these symptoms, and their sum forms a disability impairment score (FIC disability) [10]. The higher the FIC score, the greater the symptomatology (FIC-symptom scale) or the greater the disability (FIC-disability scale) [10].

### 2.4. Statistical Analysis

Means (standard deviation, SD) are presented for continuous variables, whereas proportions (percentage) are presented for categorical variables. In this study, we tested the internal consistency, reliability and construct validity properties of the FIC following the COnsensus-based Standards for the selection of health Measurement INstruments (COSMIN) [17]. 

The Cronbach alpha value was used to determine internal consistency. Values between 0.7 and 0.95 reflect the good internal consistency of an instrument [18]. Item-internal consistency was used to evaluate the reliability of the FIC. A coefficient of 0.4 supports an item-internal consistency. Additionally, item-discriminant validity was supported if the correlation between an item was higher than its correlation with all other scales. Construct validity was verified with an exploratory factor analysis. As mentioned, the FIC has just been used in the original paper [10]. Accordingly, our analysis wanted to confirm if previous assumptions regarding the items from both FIC-symptoms and FIC-disability factors are valid in a sample of COVID-19 survivors. To examine the construct validity and appropriateness of the data for factor analysis, the Kaiser–Meyer–Olkin (KMO) measure of sampling adequacy and the Bartlett test of specificity were used to determine whether the data were appropriate for factor analysis, both of which yielded values indicating the adequacy of the exploratory factor analysis. Principal component factor analysis was used to identify the number of latent factors underlying the correlations among sets of items, based on the minimum criterion of the eigenvalue of each individual factor >1. We decided to adopt a two-factor model to represent the two main constructs of the FIC: symptoms or disability-related domains. 

Finally, chi-square tests were conducted to determine differences by gender and age (grouped as <45 years, 45–59 years, 60–69 years and ≥70 years). Statistical analysis was performed with SPSS-software 23.0 (SPSS Inc, Chicago, IL, USA). Statistical significance was defined as an a priori *p*-value < 0.05.

## 3. Results

### 3.1. Participants

From 2000 patients randomly selected from the involved hospitals, a total of 1969 (mean age: 61, SD: 16 years, 46.4% women) were finally included. The reasons for exclusion can be found elsewhere [11,12,13,14,15,16]. The most prevalent onset symptoms presented by the patients at hospital admission consisted of fever (74.6%), dyspnea (31.5%) and myalgia (30.7%). 

Each patient reported a mean of 2.2 (SD 0.8) onset symptoms at hospital admission and a mean of 1.9 (SD 1.4) post-COVID symptoms at the time of study (mean: 8.4; SD, 1.5 months after hospital discharge). The features of the sample are summarized in Table 1 [11,12,13,14,15,16].

### 3.2. General Data

Each participant needs a mean of 62 (SD 11) seconds for fulfilling the FIC. No patient reported that any question was misunderstood, and all questions were perceived as comprehensible by all participants. The mean FIC symptom score was 5.35 (SD 2.9), whereas the mean FIC disability score was 3.70 (SD 2.75). The percentage of data at the ceiling (nil dysfunction) ranged from 2.29% to 9.02% while the percentage of data at the floor (maximal dysfunction) ranged from 38.56% to 80.19% (Table 2).

**Figure 1:**
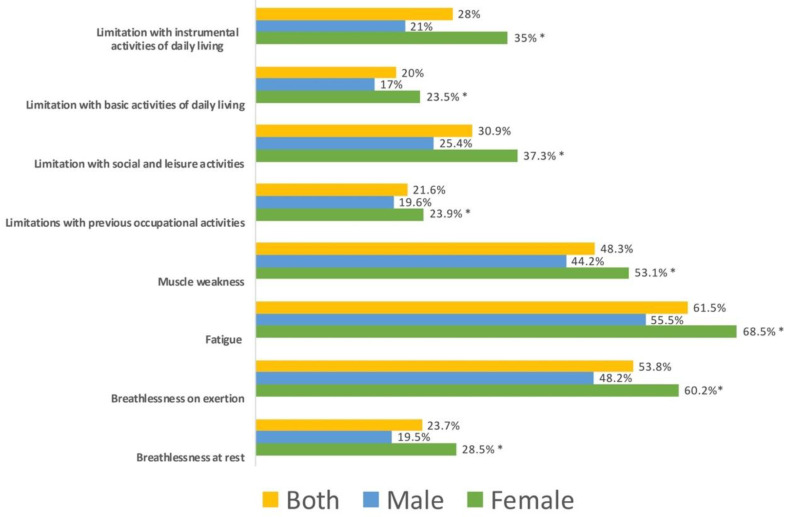
Distribution of the percentage of women and men exhibiting limitations on each item of the Functional Impairment Checklist (FIC). * Significant differences between men and women (*p* < 0.01).

**Figure 2:**
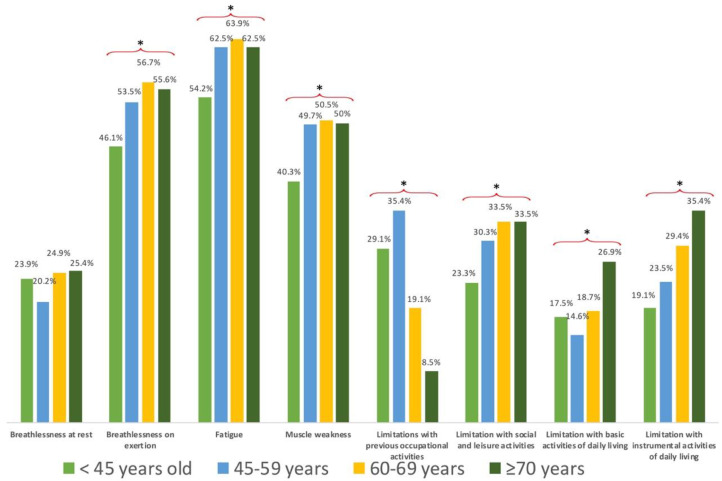
Distribution of the percentage of individuals exhibiting limitations on each item of the Functional Impairment Checklist (FIC) by age group. * Significant differences by age group (*p* < 0.01).

Women exhibited greater limitations in all physical symptoms (breathlessness at rest, *X*^2^: 22.327, *p* = 0 < 0.001; breathlessness on exertion, *X*^2^: 28.475, *p* = 0 < 0.001; fatigue, *X*^2^: 35.095, *p* = 0 < 0.001; muscle weakness, *X*^2^: 15.543, *p* < 0.001) and disability-related domains (limitations with occupational activities, *X*^2^: 5.329, *p* = 0.02; limitation with social and leisure activities, *X*^2^: 32.145, *p* < 0.001; limitation with basic activities of daily living, *X*^2^: 12.985, *p* < 0.001; limitation with instrumental activities of daily living, *X*^2^: 51.711, *p* < 0.001) of the FIC when compared with men (Figure 1). Similarly, significant differences across the age groups were observed for all domains (breathlessness on exertion, *X*^2^: 10.602, *p* = 0.014; fatigue, *X*^2^: 9.205, *p* = 0.027; muscle weakness, *X*^2^: 10.533, *p* = 0.015; limitations with occupational activities, *X*^2^: 131.58, *p* < 0.001; limitation with social and leisure activities, *X*^2^: 12.521, *p* = 0.006; limitation with basic activities of daily living, *X*^2^: 28.900, *p* < 0.001; limitation with instrumental activities of daily living, *X*^2^: 35.333, *p* < 0.001), except for breathlessness at rest (*X*^2^: 4.872; *p* = 0.181). Overall, younger patients (those aged <45 years) self-reported lower physical symptoms and disability-related domains than older patients (Figure 2).

### 3.3. Reliability and Internal Consistency

The item-internal consistency ranged from 0.766 to 0.893 for symptom-related items and from 0.695 to 0.891 for disability-related items (Table 2). Furthermore, the correlation of each of the items with its hypothesized scale was greater than its correlation with the other scale, supporting item-discriminant validity. The reliability coefficients were higher than the correlation between the FIC symptom and FIC disability scores (r: 0.676). The Cronbach’s α value of the FIC symptoms was 0.864, whereas the Cronbach’s α of the FIC disability was 0.845, supporting good internal consistency (Table 2).

### 3.4. Construct Validity

Table 3 details the results outputted from the exploratory factor analysis with varimax rotation on two factors. The eigenvalue of the first factor was 3.87, which explained 48.3% of the total measured variance, while the second factor was 1.05, which explained 13.1% of the variance. Those four items associated with disability were loaded on the first factor, with factor loadings of 0.514–0.866. Those four symptoms-associated items formed the second factor, with factor loadings of 0.603–0.832.

## 4. Discussion

The long-lasting presence of post-COVID symptoms is associated with a decrease in function and worse HRQoL [4]. The heterogeneity manifestations of long-COVID need a complete assessment covering functionality, disability and HRQoL. The PCFS was developed as a disease-specific PROM evaluating the physical functionality of COVID-19 survivors [8]; however, this PROM only covers one aspect of the disease. The FIC was originally developed as a disease-specific PROM for assessing the functional consequences of SARS [10]. This study evaluated the psychometric properties of the FIC in a large sample of COVID-19 survivors who had been previously hospitalized and exhibit long-term post-COVID symptoms. The data revealed that the FIC was internally consistent and reliable, has high degree of construct validity and showed appropriate floor and ceiling effects. These psychometric data would permit the use of the FIC for assessing symptoms and disability-related domains in COVID-19 survivors with long-COVID. Further, the FIC can be considered as a comprehensive disease-specific PROM for patients with long-COVID since it takes just 60 s to be fulfilled.

The FIC was originally designed to focus on physical symptoms (e.g., fatigue and breathlessness) and physical disability, omitting the psychological status in people who had survived SARS [10]. Lam et al. reported a Cronbach’s α coefficient of 0.75 for the FIC-symptoms scale and of 0.86 for the FIC-disability scale [10], values similar to those observed in COVID-19 survivors. These authors calculated the test-retest reliability of the FIC in a small sample of 23 patients with SARS and showed a moderate test-retest reliability (ICC 0.49–0.57) [10]. Test-retest reliability was not calculated in the current study. Our study supports the idea that the FIC could be used for people with long-COVID for assessing physical symptoms and disability-related domains, but not the emotional aspects of the condition. In fact, our large sample of patients with long-COVID experienced at least two long-lasting symptoms eight months after hospital discharge. The most common post-COVID symptoms were fatigue and dyspnea, as previously reported [3]. Interestingly, most items from the FIC-symptoms scale asked for these two physical symptoms. Therefore, other PROMs assessing emotional and psychological aspects, e.g., the Hospital Anxiety and Depression Scale (HADS), should be used in conjunction with the FIC when evaluating patients with long-COVID. Similarly, different PROMs assessing HRQoL, e.g., EQ-5D-5L or SF-36, have been previously used in individuals with post-COVID symptoms [13]. All studies reported that people with long-COVID exhibit reduced HRQoL [19]. Any of these generic PROMs may be also used in conjunction with the FIC for evaluating this other aspect of the condition. In fact, the evaluation of individuals with long-COVID should combine the information obtained from disease-specific PROMs, e.g., PCFS or the FIC, with the information obtained from generic PROMs, e.g., SF-36.

Women exhibited more symptoms and limitations than men, supporting the current assumption that the female sex is a risk factor associated with more long-term post-COVID-related symptoms [14,20]. We have also seen that older age was associated with more physical symptoms, e.g., fatigue and breathlessness, and more limitations on daily life activities. Older age is suggested in the literature as another risk factor for more disability and long-COVID symptoms, although the results are not conclusive [21,22]. In fact, the presence of a higher number of comorbidities in older people could also explain the presence of more physical symptoms, although this cofounder was controlled in our study.

These data should be understood considering their potential strengths and limitations. The inclusion of a large sample of previously hospitalized COVID-19 survivors from different centers and with a long-term follow-up period after hospital discharge is the main strength of the study. However, this is also the main limitation: the inclusion of just hospitalized COVID-19 survivors. We do not know if the FIC would exhibit similar psychometric data in COVID-19 survivors who did not require hospitalization. The second limitation is the cross-sectional design of the study, since we did not have data from these patients from before the infection or during the first months after hospitalization. It is probable that functional limitations improved with time, and the values obtained eight months after infection could be slightly better than those obtained during the first weeks or months after hospitalization. Future studies investigating the longitudinal evolution of functional disability to identify the potential responsiveness to changes in the FIC in these individuals are now needed. Finally, we did not include any objective measure of physical functioning (e.g., hand grip) or a measure of pulmonary function that could be related to functional limitations assessed by the FIC. In fact, no specific cut-off values of FIC scores have been associated with more or less symptomatology or disability-related domains. Future studies would help to elucidate these research questions. 

## 5. Conclusions

This study suggests that the FIC questionnaire exhibits good psychometric properties to be used as a disease-specific PROM to assess physical symptoms and disability-related domains in previously hospitalized COVID-19 survivors with long-COVID. The FIC is able to assess some long-COVID symptoms and should be used in combination with other PROMs.

## Figures and Tables

**Table 1:** Clinical/Hospitalization Data and Post-COVID Symptoms (n = 1969).

Variable	Data
Age, mean (SD), years	61 (16)
Gender, male/female (%)	1054 (53.5%)/915 (46.5%)
Weight, mean (SD), kg	75 (15)
Height, mean (SD), cm	165 (16.5)
Medical co-morbidities	
Hypertension	514 (26.1%)
Diabetes	236 (12.0%)
Cardiovascular Disease	234 (11.9%)
Asthma	126 (6.4%)
Obesity	88 (4.5%)
Chronic Obstructive Pulmonary Disease	77 (3.9%)
Stroke	38 (2.0%)
Rheumatological Disease	31 (1.6%)
Other (Cancer, Kidney Disease)	332 (16.9%)
Symptoms at hospital admission	n (%)
Fever	1469 (74.6%)
Dyspnoea	620 (31.5%)
Myalgia	604 (30.7%)
Cough	549 (27.9%)
Headache	332 (16.9%)
Diarrhoea	210 (10.7%)
Anosmia	167 (8.5%)
Ageusia	145 (7.35%)
Throat Pain	102 (5.2%)
Vomiting	55 (2.8%)
Stay at the hospital, mean (SD), days	11.3 (11.4)
Intensive Care Unit (ICU) admission	
Yes/No, n (%)	130 (6.6%)/1839 (93.4%)
Persistent post-COVID symptoms, n (%)	
Fatigue	1206 (61.3%)
Dyspnoea at Exertion	1054 (53.5%)
Pain Symptoms	887 (45.1%)
Loss of Hair	470 (23.9%)
Dyspnoea at Rest	459 (23.3%)
Memory Loss	341 (17.3%)
Skin Rashes	236 (12.0%)
Brain Fog	189 (9.6%)
Concentration Loss	140 (7.1%)
Tachycardia-Palpitations	140 (7.1%)
Gastrointestinal Disorders	133 (6.75%)
Ocular/Vision Disorders	116 (5.9%)
Anosmia	80 (4.05%)
Ageusia	53 (2.7%)
Throat Pain	50 (2.5%)
Diarrhoea	49 (2.5%)
Voice problems	35 (1.8%)

**Table 2:** Internal Consistency, Discriminant Validity and Floor and Ceiling Effect of each item of the Functional Impairment Checklist (FIC) in COVID-19 survivors experiencing long-COVID.

	Item-Internal Consistency	Item-Discriminant Validity	Cronbach α Value	Floor Effect	Ceiling Effect
Breathlessness at rest	0.766 ***	0.645 ***	0.864 (FIC symptoms)	76.61%	2.29%
Breathlessness on exertion	0.877 ***	0.764 ***		46.33%	6.16%
Fatigue (generalized weakness)	0.893 ***	0.781 ***		38.56%	9.02%
Muscle weakness	0.843 ***	0.703 ***		51.78%	6.82%
Limitations with previous occupational activities	0.695 ***	0.476 ***	0.845 (FIC disability)	78.68%	4.54%
Limitation with social and leisure activities	0.871 ***	0.747 ***		69.25%	3.97%
Limitation with basic activities of daily living	0.854 ***	0.746 ***		80.19%	2.95%
Limitation with instrumental activities of daily living	0.891 ***	0.789 ***		72.33%	3.73%

*** Statistically significant (*p* < 0.001)

**Table 3:** Exploratory Factor Analysis of each item of the Functional Impairment Checklist (FIC) in COVID-19 survivors experiencing long-COVID.

	Factor Loading	
	Factor 1	Factor 2
Breathlessness at rest	0.441	0.647
Breathlessness on exertion	0.192	0.832
Fatigue (generalized weakness)	0.373	0.708
Muscle weakness	0.336	0.603
Limitations with previous occupational activities	0.514	0.356
Limitation with social and leisure activities	0.712	0.235
Limitation with basic activities of daily living	0.882	0.120
Limitation with instrumental activities of daily living	0.866	0.145
Eigenvalue	3.87	1.05
Cumulative proportion of total sample variance explained	48.3%	13.1%

## Data Availability

Not applicable.
